# Immediate Implant Placement With Autologous Crown Provisionalization: A Digitally Guided Workflow

**DOI:** 10.21142/2523-2754-1404-2026-322

**Published:** 2026-10-01

**Authors:** Milton Villanueva Valenzuela, Carlos Zuloeta Tantaleán, Consuelo Marroquín-Soto, César-Augusto Padilla-Avalos

**Affiliations:** 1 Department of Restorative and Aesthetic Dentistry, Norbert Wiener University. Lima, Peru. laboratoriodentaldigital@hotmail.com Department of Restorative and Aesthetic Dentistry Norbert Wiener University Lima Peru laboratoriodentaldigital@hotmail.com; 2 Private Practice. Lima, Peru. cjzuloeta@gmail.com Private Practice Lima Peru cjzuloeta@gmail.com; 3 Research and Innovation Laboratory in Digital Dentistry, School of Dentistry, Universidad Científica del Sur. Lima, Peru. cmarroquins@cientifica.edu.pe cpadillaav@cientifica.edu.pe Universidad Científica del Sur Research and Innovation Laboratory in Digital Dentistry School of Dentistry Universidad Científica del Sur Lima Peru cmarroquins@cientifica.edu.pe cpadillaav@cientifica.edu.pe

**Keywords:** computer-aided design, dental implants, immediate dental implant loading, dental restoration, temporary, tooth extraction, diseño asistido por computadora, implantes dentales, carga inmediata de implantes dentales, restauración dental, provisional, exodoncia

## Abstract

**Background:**

Immediate implant placement in the esthetic zone is a predictable treatment approach when precise planning and appropriate soft tissue management are achieved. However, preserving the natural gingival contour remains challenging.

**Materials and Methods:**

A 53-year-old male patient presented with a vertical root fracture of the maxillary left central incisor confirmed by CBCT. Given the favorable socket morphology and the need to preserve soft tissue architecture, immediate implant placement with provisionalization was planned. After atraumatic extraction, a digitally guided workflow was used, integrating CBCT and intraoral scan data for prosthetically driven implant positioning. A tooth-supported surgical guide was fabricated to guide the osteotomy, and a GM 4.2 × 11.3 mm implant was placed with an insertion torque of 45 Ncm. The extracted clinical crown was adapted onto a temporary abutment and relined with flowable composite resin. A 3D-printed positioning guide was used to ensure accurate seating of the provisional restoration without occlusal contact.

**Results:**

The digitally guided workflow enabled accurate implant placement and immediate provisionalization using the autologous crown, preserving peri-implant soft tissue contours. Favorable early healing and stable esthetic outcomes were observed at 1 week.

**Conclusion:**

The integration of digital planning with anatomical provisionalization using the extracted clinical crown enabled predictable immediate rehabilitation in the esthetic zone. This hybrid workflow represents a simplified and patient-centered approach for achieving favorable esthetic and functional outcomes.

## RESUMEN

**Antecedentes:**

La colocación inmediata de implantes en la zona estética es un tratamiento predecible cuando se logra una planificación precisa y un manejo adecuado de los tejidos blandos. Sin embargo, preservar el contorno gingival natural sigue siendo un desafío.

**Materiales y métodos:**

Un paciente varón de 53 años presentó una fractura radicular vertical del incisivo central superior izquierdo, confirmada mediante CBCT. Dada la morfología favorable del alvéolo y la necesidad de preservar la arquitectura de los tejidos blandos, se planificó la colocación inmediata de un implante con provisionalización. Tras la extracción atraumática, se empleó un flujo de trabajo guiado digitalmente, integrando datos de CBCT y escaneo intraoral para el posicionamiento protésico del implante. Se fabricó una guía quirúrgica soportada por el diente para guiar la osteotomía, y se colocó un implante GM de 4,2 × 11,3 mm con un torque de inserción de 45 Ncm. La corona clínica extraída se adaptó a un pilar temporal y se rebasó con resina compuesta fluida. Se utilizó una guía de posicionamiento impresa en 3D para asegurar el asentamiento preciso de la restauración provisional sin contacto oclusal.

**Resultados:**

El flujo de trabajo guiado digitalmente permitió una colocación precisa del implante y una provisionalización inmediata mediante la corona autóloga, y así se preservaron los contornos de los tejidos blandos periimplantarios. Se observó una cicatrización temprana favorable y resultados estéticos estables a la semana.

**Conclusión:**

La integración de la planificación digital con la provisionalización anatómica utilizando la corona clínica extraída permitió una rehabilitación inmediata predecible en la zona estética. Este flujo de trabajo híbrido representa un enfoque simplificado y centrado en el paciente para lograr resultados estéticos y funcionales favorables.

## INTRODUCTION

Immediate implant placement in the anterior maxilla is a well-documented approach to shorten treatment duration, preserve hard and soft tissues, and improve patient satisfaction with esthetic outcomes ^1^. However, its predictability depends on careful case selection, comprehensive clinical evaluation, and prosthetically driven planning. Trauma-induced root fractures, such as in the present case, often result in sudden tooth loss and significant esthetic challenges ^2^.

The integration of CBCT and intraoral scanning has substantially improved the precision of implant placement, enabling fully guided surgery aligned with prosthetic planning ^3^^-^^5^. Immediate provisionalization plays a critical role in supporting peri-implant papillae and soft tissue contours, promoting optimal healing in the esthetic zone ^6^^−^^8^. According to recent ITI consensus reports, immediate implant placement and loading should be based on strict clinical criteria, including primary stability and socket integrity, to ensure predictable outcomes ^1^^,^^2^. In this context, digitally guided workflows contribute to improved diagnostic accuracy and prosthetically driven planning, facilitating more controlled and reproducible clinical decision-making. According to Kan et al., when combined with flapless approaches and grafting as indicated, immediate implant placement with provisionalization demonstrates reliable preservation of gingival and bony architecture ^9^.

Conventional fabrication of temporary crowns can be labor-intensive and costly. An appealing alternative is the use of the patient’s extracted clinical crown as a provisional, which provides ideal morphology, shade match, and an accurate emergence profile without the need for CAD/CAM design or milling. Clinical reports have shown that this approach also promotes soft tissue healing and papilla preservation while streamlining the workflow ^10^^,^^11^.

Complementing these analog methods, digital CAD/CAM protocols, including crown-root matrix fabrication, provide highly precise replication of natural anatomy and consistent peri-implant contours ^12^^,^^13^. Vargas et al. further introduced an indirect guided protocol for manufacturing custom provisional crowns post-extraction, emphasizing superior fit and esthetic finish ^14^.

From a clinical outcome standpoint, De Bruyn et al. reported similar survival rates between immediate and healed ridge implant placements, along with a mean bone gain of 1.6 mm and stable soft tissues over three years, confirming the long-term reliability of immediate provisional strategies ^15^.

Building on these concepts, the present case report describes a digitally guided workflow for immediate implant placement using the extracted clinical crown as an anatomical provisional. This approach aims to preserve peri-implant soft tissue architecture while integrating biological and digital principles into a simplified and reproducible clinical protocol.

## MATERIALS AND METHODS

### Diagnostic Assessment and Treatment Planning

A 53-year-old male patient presented with discoloration and discomfort in the maxillary left central incisor (#9), with a history of dental trauma. Clinical examination raised suspicion of pulp necrosis, which was confirmed via cone-beam computed tomography (CBCT) (Planmeca ProMax 3D Mid; voxel size: 75 µm), revealing a vertical root fracture and localized buccal bone loss. The imaging was processed using Romexis 6.4 software (Planmeca) (Figures 1A, 1B). Based on the radiographic and clinical findings, endodontic retreatment and apical surgery were ruled out, and a treatment plan was established for atraumatic extraction followed by immediate implant placement and provisionalization.

Digital planning was initiated by acquiring an intraoral scan of the maxillary arch using Medit i700 (70 FPS; 10.9 µm accuracy). Subsequently, DICOM data from the CBCT were exported and merged with the STL file in Blue Sky Plan 4.12.13 (BlueSkyBio) to perform prosthetically driven implant planning along the ideal axis (Figure 1B).

**Figure 1:**
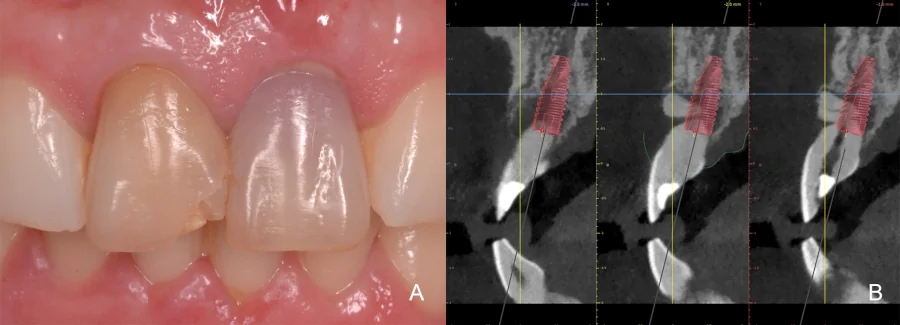
Clinical and tomographic diagnosis with virtual implant planning. A) Intraoral view of maxillary left central incisor with discoloration and translucency loss. B) CBCT cross-sectional image showing vertical root fracture and associated bone loss.

The extracted tooth presented an intact coronal structure with optimal morphology and preserved enamel integrity, making it suitable for reuse as an anatomical provisional restoration. Its selection prioritized maintenance of the patient’s natural emergence profile, contour precision, and shade continuity, following internal conditioning and disinfection procedures.

### Digital Workflow and Surgical Guide Fabrication

A tooth-supported surgical guide was designed within the same software, ensuring precise correlation with the patient’s anatomy. The guide was fabricated using a Phrozen Sonic Mini 8K S 3D printer and autoclavable Dental SG resin (JamgHe), allowing high-resolution output for accurate intraoral fit (Figures 2A, 2B).

**Figure 2:**
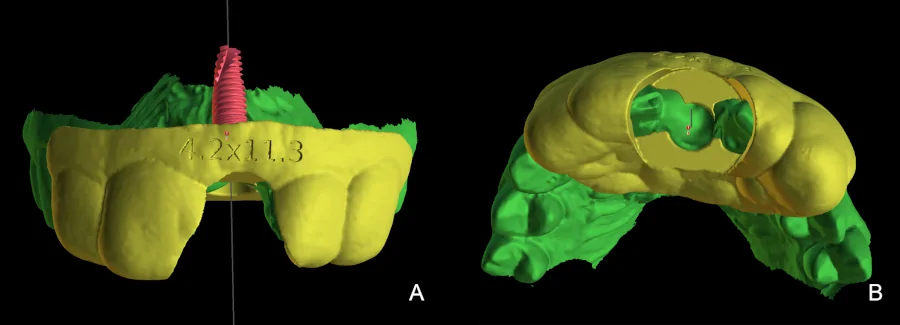
Digitally designed surgical guide. A) Buccal view showing implant trajectory aligned with prosthetic axis. B) Occlusal view showing guide sleeve position for implant placement.

### Atraumatic Tooth Extraction and Implant Surgery

Under buccal and palatal infiltration anesthesia, tooth #9 was extracted atraumatically using periotomes (Hu-Friedy) to preserve the integrity of the socket walls. Careful debridement of the socket was performed to eliminate granulation tissue and reduce bacterial load (Figures 3A, 3B).

**Figure 3:**
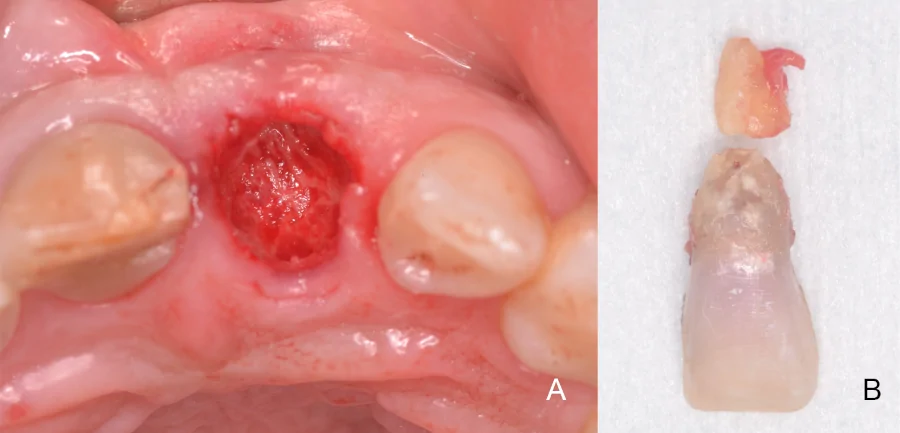
Extraction site and tooth. A) Post-extraction socket with intact soft tissue margins. B) Extracted maxillary left central incisor prepared for provisionalization.

The surgical guide was seated over the dentition, and osteotomies were performed in sequence using the Neodent surgical system. A GM 4.2 × 11.3 mm dental implant (Neodent, Straumann Group) was inserted with an insertion torque of 45 Ncm, indicating optimal primary stability for immediate provisionalization (Figures 4A, 4B).

**Figure 4:**
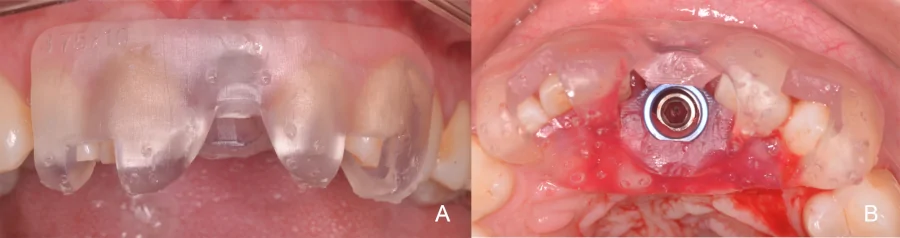
Guided implant placement. A) Tooth-supported surgical guide seated before osteotomy. B) Occlusal view of implant inserted through guide sleeve.

### Socket Grafting and Soft Tissue Management

To stabilize the peri-implant tissues and prevent buccal plate collapse, a medium-grain xenograft (Tissum) was packed into the socket gap. No attempt was made to achieve full flap coverage; rather, soft tissues were adapted and stabilized with 5-0 polyglycolic acid sutures (Tagum) to allow transmucosal healing (Figures 5A, 5B). 

**Figure 5:**
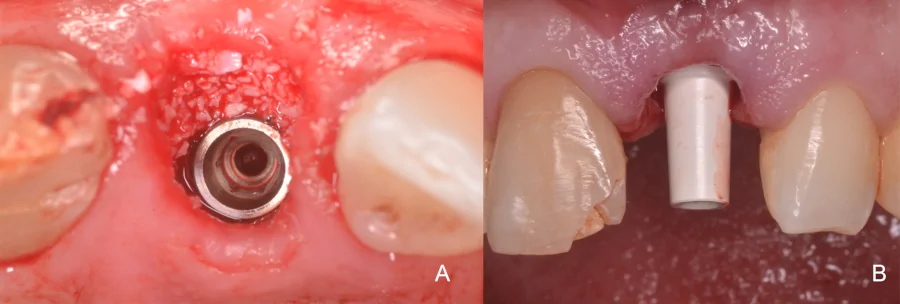
Implant placement and abutment try-in. A) Occlusal view showing implant with xenograft in socket gap. B) Temporary abutment in position, showing emergence profile.

Given the patient’s thin gingival phenotype, a connective tissue graft was indicated and performed simultaneously with the socket graft to enhance soft tissue thickness and contour stability. The graft, combined with the xenograft filling, provided adequate volumetric support for the peri-implant tissues, promoting optimal healing and esthetic integration.

### Preparation of the Extracted Crown

The clinical crown of the extracted tooth was disinfected and sectioned from the root using a bullet-shaped diamond bur under water cooling. Pulp remnants were removed, and the internal chamber was hollowed to fit a Pro Peek GM temporary abutment (Neodent). The crown was adjusted for passive seating and a supragingival finish line. Morphological adjustments were minimal to preserve the natural emergence profile (Figure 6A).

**Figure 6:**
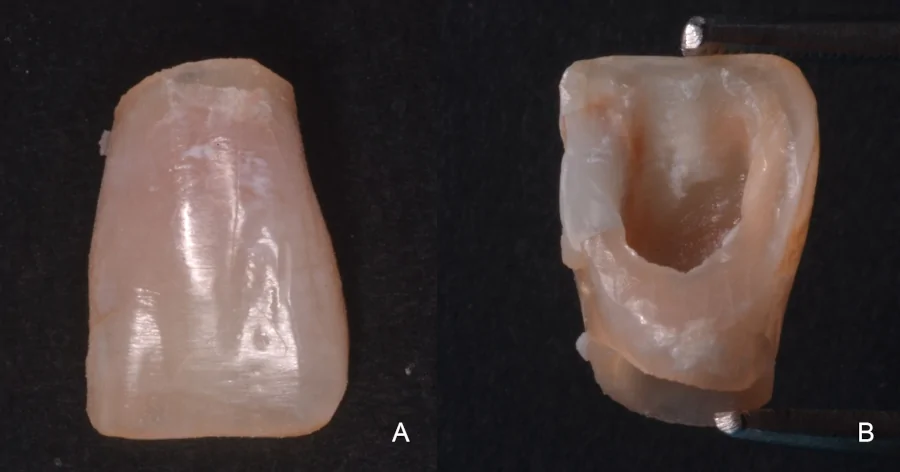
Anatomical crown adaptation and provisionalization. A) External view of extracted crown. B) Internal view after hollowing for passive adaptation to temporary abutment.

### Repositioning and Immediate Provisionalization

The crown was relined and bonded to the temporary abutment using flowable composite resin (Filtek Supreme Flowable, 3M ESPE). Light curing was completed on all surfaces for 40 seconds using a VALO Grand unit (Ultradent). Excess resin was removed, and polishing was performed with OptraGloss (Ivoclar Vivadent). The provisional was seated on the implant with a light manual torque (<10 Ncm), ensuring the absence of occlusal contacts (Figure 6B).

A digitally printed positioning guide was fabricated based on the pre-extraction digital model and post-implant planning data. This guide served as a spatial reference for repositioning the autologous crown at the exact 3D coordinates corresponding to the original tooth. It ensured accurate vertical, buccolingual, and rotational alignment during cementation, minimizing errors associated with manual repositioning (Figure 7A). The chronological sequence of the clinical workflow, including treatment stages and healing intervals, is summarized in Table 1.

**Figure 7:**
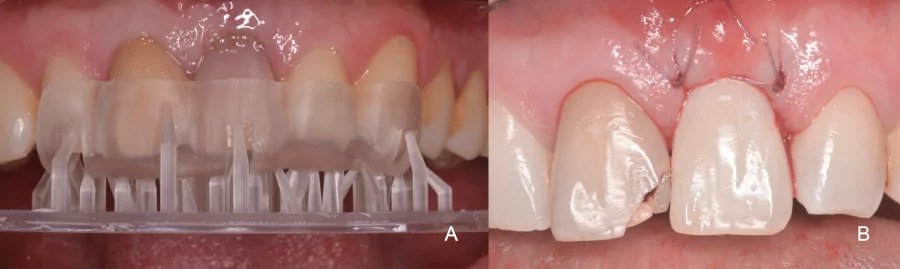
Positioning and clinical evaluation of provisional crown. A) Digitally printed positioning guide for crown repositioning. B) One-week postoperative view showing soft tissue stability.

**Table 1:** Chronological sequence of the clinical workflow

Phase	Clinical stage	Timing
Diagnostic	CBCT acquisition, intraoral scanning, digital planning	Baseline
Surgical	Atraumatic extraction, implant placement, GBR, CTG, healing abutment	Day 1
Provisionalization	Immediate provisionalization with autologous crown	Day 1
Healing	Postoperative control; soft tissue maturation and emergence profile conditioning	1-8 weeks
Digital workflow	Digital scanning of conditioned tissues	24 weeks
Final rehabilitation	Definitive implant-supported restoration placement	24 weeks

## RESULTS

At the one-week follow-up, peri-implant soft tissues demonstrated favorable healing, with preservation of marginal contours and papillae (Figure 7B). Following an 8-week soft tissue maturation period, a new intraoral scan was performed, coinciding with complete osseointegration confirmed radiographically (Figure 8A). Subsequently, the final prosthesis was fabricated with individualized emergence profiles (Figure 8B) and delivered with high esthetic integration (Figure 8C). The workflow demonstrated clinical feasibility for integrating natural morphology with digital precision in cases where flapless and biologically conservative procedures are preferred.

**Figure 8:**
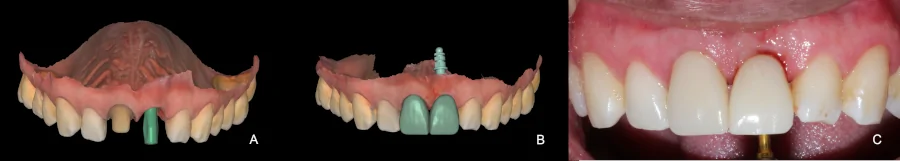
Digital workflow and final outcome. A) Intraoral scan. B) CAD design of definitive crowns. C) Final smile showing esthetic integration.

The patient expressed satisfaction with the immediate esthetic outcome, highlighting comfort and confidence during the provisional phase. He valued the reuse of his autologous crown, reporting that the approach felt natural and exceeded his expectations.

## DISCUSSION

Immediate implant placement in the anterior maxilla poses clinical challenges, particularly in preserving peri-implant soft tissue contours and recreating a natural emergence profile ^2^^,^^7^). The present technique integrates a fully guided surgical approach with immediate provisionalization using the extracted clinical crown, providing a biologically conservative, efficient, and reproducible workflow.

Prosthetically driven implant planning, based on CBCT and intraoral scan fusion, has consistently been shown to improve surgical accuracy, reduce complications, and support optimal positioning within the available bone envelope ^3^^−^^5^. In this case, the tooth-supported surgical guide ensured correct angulation and depth of implant insertion while minimizing the risk of buccal plate compromise. The achieved insertion torque (45 Ncm) guaranteed primary stability for immediate provisionalization, which was further supported by the placement of a medium-grain xenograft to stabilize the buccal socket contour. Such measures are aligned with current consensus recommendations, which emphasize atraumatic extraction, flapless surgery, and gap grafting to optimize esthetic and biological outcomes ^16^.

From a decision-making perspective, current ITI consensus reports emphasize that immediate implant placement with provisionalization is a predictable approach when adequate primary stability and favorable anatomical conditions are achieved ^1^^,^^7^. In this context, the use of digitally guided workflows enhances treatment planning accuracy and supports the execution of immediate loading protocols under controlled clinical conditions.

The reuse of the extracted clinical crown as a provisional restoration offers several unique advantages. It maintains the natural cervical morphology, texture, and shade match without the need for additional CAD design or prefabricated shells ^10^^,^^11^. This becomes particularly valuable in the esthetic zone, where the emergence profile is decisive for the final outcome ^6^^,^^8^. In the present report, the extracted crown was disinfected, hollowed, and adapted onto a GM temporary abutment, then relined with flowable composite resin. To enhance precision and reproducibility, a digitally printed positioning guide was fabricated, allowing three-dimensional control during provisional reinsertion. This step addressed one of the main limitations of analog techniques namely, accurate repositioning by incorporating digital verification into the workflow.

Studies have demonstrated the feasibility of using autologous crowns for immediate provisionalization, reporting favorable papilla preservation and soft tissue adaptation ^(10, 11)^. More recently, CAD/CAM-based strategies such as the crown-root form technique ^12^ and the autologous crown digital workflow have introduced precision manufacturing into this concept, facilitating predictable emergence profile design ^13^. In addition, guided indirect approaches for custom provisional fabrication, as proposed by Vargas et al., highlight the trend toward more standardized, esthetically refined, and reproducible outcomes ^14^.

From a long-term perspective, the stability of immediate provisionalization has also been validated in multicenter prospective studies. De Bruyn et al. reported three-year survival rates above 94% for immediate implants in both extraction and healed sites, with minimal papilla recession and an average bone gain of 1.6 mm ^15^. Similarly, Slagter et al. demonstrated that immediate and delayed provisionalization protocols in the esthetic zone yielded comparable marginal bone stability after five years, reinforcing the safety of the immediate approach ^17^.

Emerging evidence further suggests that socket integrity may not always be a limiting factor; a study showed comparable buccal bone thickness, mucosal stability, PES scores, and implant survival in intact and non-intact sockets up to ten years ^18^. Moreover, the precision of digitally guided surgery has been supported by Pitman et al., who confirmed that deviations in 3D-printed guides remain within clinically acceptable ranges (< 0.5 mm, ≤ 1°), validating their clinical reliability in immediate implant protocols ^19^.

The present protocol was carefully selected based on diagnostic and clinical criteria: the fractured tooth had no mobility, adjacent teeth were periodontally stable, and CBCT confirmed favorable socket morphology. By combining flapless atraumatic extraction, digital surgical guidance, and an anatomical provisional derived from the autologous crown, surgical trauma was minimized and peri-implant tissue preservation maximized.

Although the use of autologous crowns as provisionals has been previously described, ^16^^,^^20^^−^^22^ the proposed workflow incorporates a digital component that enhances precision and reduces variability ^23^^,^^24^. Recent evidence supports that computer-assisted implant surgery achieves high accuracy and predictable outcomes when guided by prosthetic planning ^25^. This integration offers a simplified and biologically driven approach for anterior implant rehabilitation.

Alternatively, delayed implant placement could have been considered; however, it is associated with longer treatment time and may require additional soft tissue management. Current ITI consensus reports indicate that both immediate and delayed protocols can achieve comparable outcomes when proper case selection is ensured ^1^. In this case, immediate placement was selected due to favorable socket morphology and primary stability, enabling a conservative and esthetically driven approach.

This report presents a single clinical case and cannot be generalized. Further prospective studies, including the evaluation of peri-implant healing parameters such as probing depth and bleeding on probing, are required to confirm the reproducibility and long-term performance of this digitally guided protocol.

## CONCLUSIONS

The present report describes a digitally guided workflow for immediate implant placement using the extracted clinical crown as an anatomical provisional. This approach enables precise implant positioning, preservation of peri-implant soft tissues, and predictable esthetic outcomes. It may represent a practical and reproducible alternative for anterior implant rehabilitation.
